# Targeted Versus Nontargeted Communication About Electronic Nicotine Delivery Systems in Three Smoker Groups

**DOI:** 10.3390/ijerph15102071

**Published:** 2018-09-21

**Authors:** Bo Yang, Jiaying Liu, Lucy Popova

**Affiliations:** 1 Tobacco Center of Regulatory Science, School of Public Health, Georgia State University, P.O. Box 3995, Atlanta, GA 30302, USA; byang12@gsu.edu; 2 Department of Communication Studies, University of Georgia, Athens, GA 30602, USA; jiaying.liu@uga.edu

**Keywords:** audience segmentation, ENDS, message targeting, cigarettes, anti-smoking communication

## Abstract

*Background:* This study used an audience segmentation and message targeting approach to identify three distinct smoker groups—Older Freedom Smokers (OFS), Reluctant Smokers (RS), and Young Enthusiasts (YE)—and examined whether an electronic nicotine delivery systems (ENDS) message targeting each smoker group (targeted message) was associated with more health-enhancing outcomes than messages targeting other groups (nontargeted messages). *Methods:* An online experiment was conducted among 580 adult smokers with 180 OFS, 200 RS, and 200 YE. Each smoker group viewed a targeted message and two nontargeted messages in a random order. Following the presentation of each message, participants reported their perceived message effectiveness, message reactions, ENDS- and cigarette-related beliefs, and behavioral intentions. *Results:* The targeted vs. nontargeted messages mostly did not produce more health-enhancing outcomes on perceptions of absolute and comparative risks of ENDS and cigarettes, response efficacy of ENDS, and self-efficacy as well as intentions to quit smoking. *Conclusions:* Our targeted messages did not appear to be a better choice over nontargeted messages to communicate about ENDS to smokers. Given the increasing call to accurately inform the public of the risk differences among various tobacco products, future studies should continue to explore whether targeted messages could be employed to communicate about the comparative risks of ENDS.

## 1. Introduction

### 1.1. Background

Electronic nicotine delivery systems (ENDS), also known as e-cigarettes or “vapes,” use battery-powered heating elements (coils) to heat up a liquid solution typically containing propylene glycol and/or vegetable glycerin, nicotine, and flavors to produce an aerosol. In 2016, 15.4% of U.S. adults had ever used ENDS [1]. While the long-term health effects of ENDS are still undetermined, ENDS emissions contain lower amounts of many harmful chemicals than combusted cigarettes [2,3].

ENDS could potentially play a role in reducing the overall harm of tobacco use if smokers unwilling to quit smoking switch to them completely [4,5,6]. However, ENDS might also have negative public health implications if they inhibit cessation in smokers who are interested in quitting. This presents a challenge of how to best communicate about ENDS, particularly their potentially lower levels of risk compared to cigarettes.

Recent years have seen an increasing misperception among the public about the risk differences between ENDS and cigarettes [7]. Because the perception that ENDS are less harmful than cigarettes is usually cited as one of the most important reason for their use [8,9], it is imperative to understand how to communicate to the public about the comparative risk of ENDS and cigarettes. However, only a few studies have examined the effects of messages communicating the risk differential between ENDS and combusted cigarettes [10,11,12,13,14,15]. These studies found that comparative risk messages about ENDS were able to reduce smokers’ perceived risk of ENDS relative to combusted cigarettes [13,14,15], increase smokers’ interest in using ENDS [10,16], and reduce smoking intentions [15] and smoking behavior during the study [17]. Despite these promising findings, two studies concluded that messages presenting lower risk information about ENDS relative to cigarettes had no effects on smoking cessation intentions [15,17]. One study found that communicating ENDS as being less harmful than cigarettes increased smokers’ interest in using both ENDS and cigarettes [13]. These findings suggested that comparative risk communication about ENDS may raise some unintended problems and that more research is needed to identify communication strategies that can foster positive and minimize negative outcomes. Particularly, attention should be paid to evaluating different approaches to communicating about ENDS by considering smokers’ characteristics as smokers vary substantially in their attitudes, risk perceptions, and use experiences with ENDS and cigarettes, and these differences may lead to preferences for different types of health risk messages [18]. For instance, young adults are more likely to use ENDS and perceive ENDS as being less harmful than cigarettes [7,19,20]. In contrast, older adults are more likely to use traditional cigarettes and are less aware of ENDS [21,22]. In addition, older adults are more likely than young adults to regret smoking [23]. Consequently, communicating to younger vs. older smokers about ENDS may require different types of messages. Prior studies have found audience segmentation and message targeting can enhance health communication effectiveness. However, this approach was not examined in the context of comparative risk communication about different tobacco products. To fill these research gaps, our study explores whether audience segmentation and message targeting are useful strategies for communicating comparative ENDS risk to smokers.

### 1.2. Audience Segmentation and Message Targeting

In health communication, the strategic combination of audience segmentation and message customization is considered an efficient way to optimize persuasive outcomes among targeted subpopulations [24,25]. Audience segmentation is a process of dividing audiences into relatively homogeneous groups based on their demographic, cognitive, and behavioral characteristics [26,27,28,29]. Grunig’s [30] nested model of segmentation proposed inner (i.e., individual-level characteristics) and outer nests (i.e., characteristics based on individuals’ membership in larger social groups) as in concentric circles to guide segmentation practices. Drawn substantially from theories of behavioral change, Slater and Flora [31] further developed a framework to partition individuals based on three sets of key determinants of health behavior: cognitive (e.g., attitude, involvement, risk perceptions, self-efficacy perceptions), social influence (e.g., reference group norms, social expectations, visibility of behavior), and personal history (e.g., individuals’ current and previous behavior practices). Both models consider individual-level characteristics as the most useful audience segmentation variables as these variables provide the most specific and direct information about the target population, permitting more precise segmentation [32]. Groups produced through audience segmentation usually differ vastly in the characteristics related to the communication outcomes of interest and hence are expected to prefer different types of messages, which introduces the concept of message targeting [25].

Compared with nontargeted health messages, targeted messages are considered to be able to produce more desirable health behavior changes [25]. This persuasive advantage could be explained by both information processing theories (e.g., elaboration likelihood model, or ELM) and behavioral theories [33]. According to the ELM [34], messages that appear to be self-relevant, such as targeted messages, lead to central processing, which is associated with more persistent persuasion outcomes. In addition, message targeting is usually done based on behavioral determinants identified by behavioral theories, such as social cognitive theory [35], theory of reasoned action [36], and transtheoretical model of behavior change [37]. Targeted messages incorporate characteristics of behavioral determinants unique to each group into their design and hence are more likely to cause behavioral change.

Targeted messages have previously been contrasted with two types of nontargeted messages: (1) generic or standard (one-size-fits-all) messages that provide information geared toward the general population of interest and (2) mismatched messages, which are crafted to resonate with a different subgroup in the same population (such as messages targeting smokers in a different cessation stage, e.g., showing messages targeting those in the action stage to smokers in the precontemplation stage). Comparing targeted messages with generic messages is the most common and has been examined extensively. Substantial evidence has demonstrated that targeted messages are more effective than generic ones in promoting changes across many behavior domains [24,38,39,40,41,42,43]. Existing empirical evidence comparing targeted messages with mismatched messages, however, remains scarce and mixed. For example, in one study [44], audiences were segmented into four subgroups based on their perceived risk and efficacy toward diabetes screening. Then, audiences were randomized to view a message that either matched or mismatched their group’s characteristics, or a control message. The results showed that participants viewing a matched message reported significantly greater intentions to engage in self-protective behavior than those in the mismatched or control groups. In another study [45], researchers segmented audiences based on their cultural backgrounds and found that organ donation messages targeting a given audience segment were equally persuasive as the messages targeting other segments on willingness to donate organs.

In the real-world practice of health communication, people are usually exposed to various types of health messages and may accidentally view messages targeting other audience segments. Given this, the extent to which targeted messages can be more effective than mismatched messages in changing beliefs and behaviors warrants more investigation. Thus, our current study focuses on examining the effects of ENDS targeted messages in comparison with the mismatched messages. We hereinafter refer to these comparator messages as “nontargeted messages” for simplicity.

### 1.3. The Present Study

Tobacco companies and public health agencies have been using audience segmentation and targeted messaging in tobacco-related communication. For example, tobacco companies conducted sophisticated research to segment female audiences into “job-holders”, “career women”, and “the housewives”, among other groups, and developed messages specifically targeting low-income women [46]. The U.S. Food and Drug Administration’s (FDA) “Fresh Empire” campaign targeted hip-hop youths with messages that emphasized the disconnection between hip-hop values, such as creativity, authenticity, and hard work, and the negative outcomes of tobacco use [47].

While effects of messages on comparative ENDS risks have sometimes been evaluated among separate demographic groups or by product use status [10,12,48], past research has not employed more nuanced psychographic segmentation to determine the target audiences. Informed by the aforementioned theoretical models of segmentation and the previous segmentation efforts in the tobacco domain [10,49], we took individual-level characteristics, including current and previous tobacco use behavior practices, cognitions about ENDS use, and demographic information, to be the focal segmentation parameters in the present study. Given that the evidence mostly supports the persuasive advantage of targeted communication and that smoker subgroups vary on their risk perceptions, attitudes, and behaviors surrounding ENDS and cigarettes, our main hypothesis was that (1) targeted messages about the comparative risks of ENDS and cigarettes will produce (a) lower risk perception about ENDS, (b) higher risk perception about cigarettes, (c) higher efficacy beliefs about using ENDS to quit smoking, and (d) stronger intentions to quit smoking than nontargeted messages. Risk perception, efficacy beliefs, and intentions are important predictors of actual behaviors [50,51]. Treating these variables as primary outcome variables to examine targeted vs. nontargeted messages will provide useful suggestions for tobacco control practice. Additionally, because message reactions are also commonly used to gauge message effectiveness [52], we developed the second hypothesis that (2) targeted messages about the comparative risks of ENDS and cigarettes will produce (a) higher perceived message effectiveness, (b) stronger emotional responses, (c) weaker smoking cravings (physiological responses), and (d) stronger ENDS cravings (physiological responses) than nontargeted messages. To test these hypotheses, we identified three distinctive smoker segments or groups. For each segment, we developed a targeted message. In an online experiment, we examined whether each group’s responses to the targeted message were more favorable than responses to the messages targeting other groups (i.e., nontargeted messages).

## 2. Materials and Methods

### 2.1. Audience Segmentation

We worked with a public relations agency Porter Novelli (PN) to develop and test messages. The first step was to define the characteristics that would be useful in segmenting the smokers for ENDS messaging and that would allow for construction of conceptually distinct messages about ENDS. We reviewed the extant literature on ENDS messaging, and one segmentation used in studies was categorizing people by smoking behavior into smokers and nonsmokers [10]. As our focus was on smokers only, the relevant characteristic was their use of ENDS in combination with their smoking behavior and their perceptions and intentions regarding the products. Our approach to partitioning individuals based on their tobacco use behavior status was similar to how tobacco companies segment their audience [49]. These tobacco use behaviors were examined in the data our team had collected in 2015 in a nationally representative survey of U.S. adults’ risk perceptions towards tobacco products and the data from 2016 PN Consumer Styles survey to further identify what cognitions and demographic characteristics were associated with the distinct e-cigarette and cigarette use. Based on the three focal segmentation parameters (behavioral, cognitive, and demographic), the qualitative reviews of the data identified three distinct groups. One group was currently using ENDS at least three times per week or more, viewed ENDS as a lifestyle choice and identity, and was vocal on social media about their support for ENDS. They tended to be younger and more educated. Another group was hard-set in their smoking habits, having little tolerance of others telling them what to do, had used ENDS and were currently using them once a month or less frequently, and held individualistic worldviews. They tended to be older and had lower income and education. A third group wanted to quit smoking but had not used ENDS to support their quitting attempts. They tended to be older than the first group but younger than the second. These groups were labeled Young Enthusiasts (YE), Older Freedom Smokers (OFS), and Reluctant Smokers (RS), respectively. Their characteristics are presented in Table 1 and the questions used to identify them for the survey are reported in the Supplemental Material. It should be noted that these three groups do not encompass all possible smoker groups, but they identify specific audience segments, which allows for creation of targeted messages.

PN then conducted six focus groups (two per smoker group) with 54 participants in Chicago, IL and Atlanta, GA as part of the message development (described below) and assessed the groups’ attitudes and risk perceptions about ENDS and general health beliefs (for detailed profile of each group, see Table 1).

### 2.2. Message Development

Messages in all groups sought to motivate smokers to quit smoking and to include ENDS in their quit plan while not engaging in dual use. According to theories of behavioral change [50,53], people’s cognitive beliefs and prior behavior predict their future action. Hence, the message targeting each group primarily focused on addressing the smoker group’s prior experiences with ENDS, ENDS attitudes, and risk perceptions. For instance, OFS and RS groups were less likely to hold correct perceptions about comparative risk of ENDS and use ENDS to quit smoking. On the other hand, many people in the YE group correctly understood ENDS comparative risks, were ENDS users, and were more likely to see ENDS as a way to reduce combusted cigarette use. Consequently, the message targeting OFS and RS focused more on using ENDS to quit smoking than the message targeting YE. The message targeting YE talked more about absolute ENDS risk in order to discourage dual use than the messages targeting OFS and RS.

These initially developed messages were tested in six focus groups (two per smoker group) with 54 participants in Chicago, IL and Atlanta, GA. Participants were selected based on their age, education, income, prior ENDS use experiences, and worldviews (see “Inclusion Criteria” in Table 1). Only those matching the profile of one of the smoker groups identified early were allowed to participate in the focus group discussion and were presented with the messages designed for their group. During the discussion, two focus groups of each smoker segment evaluated whether their targeted message could motivate them to quit smoking combusted cigarettes, possibly with the use of ENDS. Messages were then refined. The final message for each group is presented in Table 2.

### 2.3. Participants

Three messages were tested in an online message evaluation study among adult current smokers (18–64 years old) who had smoked 100 cigarettes or more in their lifetime and were currently smoking every day or some days. A marketing research company, Focus Pointe Global (FPG), recruited participants from their online panel and administered the survey. Before entering the experiment, participants were first screened for whether they matched the profile of any of the three smoker groups identified early (see the Supplemental Material for specific screening questions). As with the focus group discussion, only those matching a smoker group profile were allowed to proceed (see “Inclusion Criteria” in Table 1). This quantitative study included a total of 580 smokers (180 OFS, 200 RS, and 200 YE). 

The majority of OFS were female (74.4%) and White (78.9%), with an average age of 52.93 (*SD* = 5.18). Among RS, 76.5% were female and 50.5% were White. Mean age was 39.96 (*SD* = 7.30). In the YE sample, 59% were female and 67% were White, and the average age was 26.4 (*SD* = 3.07).

### 2.4. Procedure

Participants began by answering questions about their tobacco use patterns and perceptions of ENDS. Then, each participant was shown all three written messages (i.e., a message targeting their group [targeted message] and two messages targeting other groups [nontargeted messages]) in a random order. After each message, participants answered questions about their reactions to the message, including positive and negative emotions, perceived message effectiveness, perception of the risks of ENDS and cigarettes, ENDS efficacy, smoking and ENDS use cravings, and behavioral intentions. All subjects gave their electronic informed consent before they participated in the study. The study was conducted in accordance with the Declaration of Helsinki, and the protocol was approved by Georgia State University’s Institutional Review Board (IRB number: H16770).

### 2.5. Key Measures

Based on our hypotheses, the primary outcomes included risk perception about ENDS, risk perception about cigarettes, efficacy beliefs about using ENDS, and quit intentions. We also assessed perceived message effectiveness, emotional responses, and physiological responses, including smoking and ENDS use cravings, as secondary outcomes.

Risk perception about ENDS included perceived comparative and absolute risk of ENDS. Perceived comparative risk of ENDS and cigarettes was assessed with a single question: “Is using electronic vapor products less harmful, about the same, or more harmful than smoking regular cigarettes?” [54]. Participants were asked to pick one option from “much less harmful”, “less harmful”, “about the same level of harm”, “more harmful”, “much more harmful”, and “I don’t know”. Similar to prior studies [7,15,54], we combined “much less harmful” and “less harmful” into one category to represent correct comparative risk beliefs about ENDS and coded this as 1. All other answers were combined to represent a category of misperception or no knowledge about comparative ENDS risk, coded as 0. Perceived absolute ENDS risk was assessed by asking people to indicate how much they agree with the statements that using ENDS is harmful on a 5-point Likert-type scale (1 = strongly disagree, 3 = neutral, 5 = strongly agree). Both measures were also assessed before any message exposure.

Risk perception about cigarettes included perceived absolute cigarette risk. The variable was assessed both before and after message exposure. People were asked to indicate how much they agree with the statement that smoking is harmful on a 5-point Likert-type scale (1 = strongly disagree, 3 = neutral, 5 = strongly agree).

Efficacy beliefs about using ENDS included ENDS response efficacy and people’s perceived self-efficacy to quit smoking using ENDS. Response efficacy was assessed both before and after message exposure. Response efficacy [55] was assessed by two items: efficacy of ENDS to help quit smoking and efficacy of ENDS to help reduce smoking. Participants rated their agreement with each item on a 5-point Likert-type scale (1 = strongly disagree, 3 = neutral, 5 = strongly agree). Because efficacy of ENDS to help quit smoking and efficacy of ENDS to help reduce smoking are conceptually different, we did not combine the two items. Finally, self-efficacy [55] was assessed through a single question: “If you were to try to quit smoking cigarettes completely using an electronic vapor product, how likely do you think you would be to succeed?” People provided their answers on a 4-point scale (1 = not at all, 2 = a little likely, 3 = somewhat likely, 4 = very likely).

Quit intentions were measured with an item “I want to quit smoking” with responses on a 5-point Likert-type scale (1 = strongly disagree, 3 = neutral, 5 = strongly agree).

Perceived message effectiveness was measured with nine items (e.g., “This message is convincing”) on a 5-point Likert-type scale (1 = strongly disagree, 3 = neutral, 5 = strongly agree). The nine ratings were averaged into a scale (Cronbach’s α = 0.96).

Emotional responses included negative and positive emotional responses. Negative emotions were assessed through five items: angry, afraid, ashamed, sad, and regretful [56,57,58]. Ratings of the five items on a 5-point scale (1 = I do not feel this emotion, 3 = moderate emotion, 5 = extreme and intense emotion) were averaged (α = 0.94). Similarly, positive emotions [59] were assessed through two items—motivated and hopeful—on a 5-point scale, and the average was calculated to represent the scale (α = 0.89).

Physiological reactions to the messages were smoking cravings (“I want a cigarette right now”) and ENDS use cravings (“I want to use my electronic vapor product right now”) measured on a 5-point Likert-type scale (1 = strongly disagree, 3 = neutral, 5 = strongly agree).

### 2.6. Analysis Plan

As indicated earlier, our study defined nontargeted messages as the messages that targeted other groups. We compared responses to targeted and nontargeted messages in terms of ENDS and cigarette risk perception, ENDS efficacy beliefs, quit intentions, perceived message effectiveness, and emotional and physiological reactions separately for each group. 

When the outcome variables were cravings, ENDS and cigarette risk perceptions, ENDS efficacy beliefs, and behavioral intentions, people’s responses to later messages on these variables might be influenced by their prior message exposure and responses. Therefore, we analyzed responses on these variables only from the first message the participants saw. Several ANCOVAs with specified contrasts (targeted message = 2, untargeted messages = −1, respectively) were performed with message condition as a between-subject factor and perceived absolute ENDS and cigarette risks, perceived comparative risk of ENDS and cigarettes, and ENDS response efficacy measured at pretest as covariates. These pretest variables were controlled for because they might influence the actual effects of the messages on cigarette- and ENDS-related beliefs and intentions. Because comparative risk was coded into a binary variable, in order to analyze the effects of targeted vs. nontargeted messages on this variable, we performed a binominal logistic regression. The binomial logistic regression model included two dummy variables comparing the targeted message with each of the two nontargeted messages. All analyses were conducted using SPSS version 24 (IBM Corp., Armonk, NY, USA).

When the outcome variables were perceived message effectiveness or emotional reactions to the messages, we treated message condition as a within-subject factor and performed three repeated-measures ANOVAs with planned contrasts comparing the targeted message (contrast = 2) with the two nontargeted messages (contrast = −1 for each message).

## 3. Results

Our primary hypothesis was that targeted messages about the comparative risks of ENDS and cigarettes would produce (a) lower risk perception about ENDS, (b) higher risk perception about cigarettes, (c) higher efficacy beliefs about using ENDS to quit smoking, and (d) stronger intentions to quit smoking than nontargeted messages. Second, we predicted that targeted messages about the comparative risks of ENDS and cigarettes would produce (a) higher perceived message effectiveness, (b) stronger emotional responses, (c) weaker smoking cravings (physiological responses), and (d) stronger ENDS cravings (physiological responses) than nontargeted messages.

### 3.1. Perceived Comparative Risk of ENDS and Cigarettes 

The RS group was more likely to report that ENDS were less harmful than cigarettes after exposure to the targeted vs. nontargeted messages. There was no difference between targeted and nontargeted messages on comparative risk perception of ENDS for YE or OFS (Table 3). H1a was partially supported for comparative risk perception of ENDS and cigarettes.

### 3.2. Perceived Absolute Risk of ENDS 

For OFS, the omnibus test showed that message condition was significant on perceived absolute ENDS risk, *F*(2, 172) = 5.42, *p* < 0.01, η_p_^2^ = 0.06. For RS, the omnibus test indicated message condition was marginally significant on perceived absolute ENDS risk, *F*(2, 192) = 3.02, *p* = 0.05, η_p_^2^ = 0.03. However, we did not find our contrast model comparing targeted with two nontargeted messages significant for absolute risk variables for either OFS or RS. For YE, the omnibus test indicated that message condition was significant on perceived absolute ENDS risk, *F*(2, 192) = 5.40, *p* < 0.01, η_p_^2^ = 0.05. Follow-up comparisons showed that after seeing the targeted vs. two nontargeted messages, YE reported higher perceived absolute ENDS risk, *F*(1, 192) = 10.76, *p* < 0.01, η_p_^2^ = 0.05, which was opposite to what we predicted (Table 4). H1a was not supported for absolute ENDS risk perception.

### 3.3. Perceived Absolute Risk of Cigarettes 

For all groups, the omnibus tests showed that message condition was not significant on perceived absolute cigarette risk—for OFS: *F*(2, 172) = 0.51, *p* = 0.60, η_p_^2^ = 0.01; for RS: *F*(2, 192) = 0.14, *p* = 0.87, η_p_^2^ = 0.00; and for YE: *F*(2, 192) = 0.68, *p* = 0.51, η_p_^2^ = 0.01 (Table 4). H1b was not supported.

### 3.4. ENDS Efficacy Beliefs 

For OFS, the omnibus tests showed that message condition was significant on response efficacy of ENDS to help quit smoking, *F*(2, 172) = 10.19, *p* < 0.001, η_p_^2^ = 0.11, and response efficacy of ENDS to help reduce smoking, *F*(2, 172) = 9.31, *p* < 0.001, η_p_^2^ = 0.10, but was not significant on self-efficacy, *F*(2, 172) = 0.92, *p* = 0.40, η_p_^2^ = 0.01. Despite two significant omnibus tests, our predicted contrast models comparing the targeted message with two nontargeted messages were not significant for any of the efficacy variables (Table 4). 

For RS, the omnibus tests indicated message condition was significant on self-efficacy, *F*(2, 192) = 5.24, *p* < 0.01, η_p_^2^ = 0.05, and was not significant on response efficacy of ENDS to help quit smoking, *F*(2, 192) = 0.68, *p* = 0.51, η_p_^2^ = 0.01, or response efficacy of ENDS to reduce smoking, *F*(2, 192) = 1.54, *p* = 0.22, η_p_^2^ = 0.02. Again, we did not find our contrast model comparing targeted with two nontargeted messages significant for any of the efficacy variables (Table 4).

For YE, the omnibus tests indicated that message condition was significant on response efficacy of ENDS to help quit smoking, *F*(2, 192) = 8.52, *p* < 0.001, η_p_^2^ = 0.08, response efficacy of ENDS to help reduce smoking, *F*(2, 192) = 5.18, *p* < 0.01, η_p_^2^ = 0.05, but was not significant on self-efficacy, *F*(2, 192) = 2.13, *p* = 0.12, η_p_^2^ = 0.02. Follow-up comparisons showed that our predicted contrast model was significant for response efficacy of ENDS to help quit smoking, *F*(1, 192) = 16.78, *p* < 0.001, η_p_^2^ = 0.08, response efficacy of ENDS to reduce smoking, *F*(1, 192) = 10.30, *p* < 0.01, η_p_^2^ = 0.05, and self-efficacy, *F*(1, 192) = 4.12, *p* < 0.05, η_p_^2^ = 0.02. Specifically, after seeing the targeted vs. two nontargeted messages, YE reported lower response efficacy of ENDS to help quit or reduce smoking and lower self-efficacy for using ENDS to quit smoking (Table 4). Overall, the findings did not support H1c.

### 3.5. Quit Intentions 

For OFS, the omnibus test found that message condition was not significant on quit intentions, *F*(2, 172) = 0.01, *p* = 1.00, η_p_^2^ = 0.00. For RS, the omnibus test showed that message condition was not significant on quit intentions, *F*(2, 192) = 0.56, *p* = 0.56, η_p_^2^ = 0.01. For YE, the omnibus test showed that message condition was significant on quit intentions, *F*(2, 192) = 3.82, *p* < 0.05, η_p_^2^ = 0.04. The targeted message predicted lower quit intentions than the two nontargeted messages for YE, *F*(1, 192) = 4.05, *p* < 0.05, η_p_^2^ = 0.02 (Table 4). H1d was not supported.

### 3.6. Perceived Message Effectiveness 

According to the repeated measures ANOVAs, for OFS, the targeted and two nontargeted messages were rated as equally persuasive. For RS, compared with nontargeted messages, the targeted message led to higher levels of perceived message effectiveness, *F*(1, 199) = 4.62, *p* = 0.03, η_p_^2^ = 0.02. In contrast, for YE, the targeted message led to lower levels of perceived message effectiveness than the nontargeted messages, *F*(1, 199) = 24.59, *p* < 0.001, η_p_^2^ = 0.11 (Table 5). H2a was partially supported.

### 3.7. Emotional Responses

Repeated measures ANOVAs with specified contrasts found that for OFS, the targeted message led to higher levels of positive emotions than the nontargeted messages, *F*(1, 179) = 10.05, *p* < 0.01, η_p_^2^ = 0.05. For RS, the targeted message led to higher levels of both negative emotions, *F*(1, 199) = 10.38, *p* = 0.001, η_p_^2^ = 0.05, and positive emotions, *F*(1, 199) = 15.14, *p* < 0.001, η_p_^2^ = 0.07. For YE, the targeted message led to lower levels of positive emotions, *F*(1, 199) = 50.18, *p* < 0.001, η_p_^2^ = 0.20 (Table 5). H2b was partially supported.

### 3.8. Physiological Responses 

For OFS, ANCOVA omnibus tests found that message condition was significant on ENDS use cravings, *F*(2, 172) = 6.88, *p* = 0.001, η_p_^2^ = 0.07, and marginally significant on smoking cravings, *F*(2, 172) = 2.65, *p* = 0.07, η_p_^2^ = 0.03. Our contrast model comparing the targeted message with two nontargeted messages achieved significance only on ENDS use cravings, *F*(1, 172) = 5.74, *p* < 0.05, η_p_^2^ = 0.03: OFS exposed to the targeted message reported higher ENDS use cravings than OFS exposed to the two nontargeted messages (Table 5).

For RS, the omnibus test showed that message condition was significant on ENDS use cravings, *F*(2, 192) = 3.18, *p* < 0.05, η_p_^2^ = 0.03, but was not significant on smoking cravings, *F*(2, 192) = 0.66, *p* = 0.52, η_p_^2^ = 0.01. In the follow-up comparison, the targeted message vs. the two nontargeted messages did not reach significance on either craving variable (Table 5).

For YE, the omnibus tests showed that message condition was marginally significant on ENDS use cravings, *F*(2, 192) = 2.99, *p* = 0.05, η_p_^2^ = 0.03, but was not significant on smoking cravings, *F*(2, 192) = 1.25, *p* = 0.29, η_p_^2^ = 0.01. In the follow-up comparison, our contrast model was significant on ENDS use cravings, *F*(1, 192) = 4.98, *p* < 0.05, η_p_^2^ = 0.03, but was not significant on smoking cravings, *F*(1, 192) = 1.86, *p* = 0.17, η_p_^2^ = 0.01. The targeted message predicted lower ENDS use cravings than the two nontargeted messages for YE (Table 5). Overall, the findings rejected H2c and partially supported H2d.

## 4. Discussion

While ENDS use has increased exponentially in recent years, public knowledge about the risk of ENDS compared with traditional cigarettes remains limited [7]. In the context of emerging calls for clear communication about the risk differences among various tobacco products [18,60], our study segmented smokers into three distinctive groups—Older Freedom Smokers (OFS), Reluctant Smokers (RS), and Young Enthusiasts (YE)—and examined whether for each smoker group, ENDS messages targeting the group had more positive impacts than the nontargeted messages.

Findings suggest that our targeted vs. two nontargeted messages were mostly limited in producing more health-enhancing responses. For all three smoker groups, targeted and nontargeted messages led to similar perceived absolute cigarette risks. In our data, OFS, RS, and YE all reported cigarettes to be extremely harmful (Table 4). Such a highly skewed distribution may have created a ceiling effect such that both targeted and nontargeted messages served as reminders rather than persuasive appeals to enhance their already negative perceptions of cigarettes.

The advantage of the targeted message over the nontargeted messages in shaping correct perception of ENDS risk relative to cigarettes was limited to only RS. Compared with OFS and YE, RS had more concerns about the safety of ENDS (Table 1). Hence, they might have read our messages more carefully and been more likely to notice the differences between targeted and nontargeted messages in informing them of the comparative risk of ENDS compared with cigarettes.

Effects of the targeted message and two nontargeted messages differed in absolute ENDS risk perception, response efficacy, and self-efficacy only for YE. The findings revealed that YE exposed to targeted vs. two nontargeted messages reported higher levels of perceived absolute ENDS risk, lower levels of response efficacy of ENDS to help quit or reduce smoking, and lower levels of self-efficacy for using ENDS to quit smoking. These beliefs might indicate a lower chance of completely switching to ENDS to reduce current health risks among YE smokers who are unwilling to quit smoking. But these beliefs are positive from the perspective of eliminating any tobacco use.

Finally, we found OFS and RS exposed to the targeted vs. two nontargeted messages reported no differences between the messages on quitting intentions. YE reported lower quit intentions following targeted vs. two nontargeted messages. Hence, for all three smoker groups, targeted messages were not more persuasive than nontargeted messages in encouraging smoking cessation. In addition, OFS reported greater ENDS use cravings following the targeted vs. two nontargeted messages, which might indicate that targeted messages worked better in motivating them to use ENDS. However, because targeted messages did not differ from nontargeted messages in quit intentions for OFS, it is uncertain whether targeted vs. nontargeted messages will motivate OFS to engage in dual use in the long run.

Public health communication to smokers about ENDS should help smokers correctly understand the risks of ENDS relative to cigarettes and encourage smokers to quit smoking or to switch completely to ENDS. However, our study mostly did not find that our targeted vs. nontargeted messages were more likely to achieve these goals. Hence, perhaps the right set of targeted messages exists, but our targeted messages were not more effective than nontargeted messages to communicate about comparative ENDS risks. Essentially, effective targeted communication should be based on sensible audience segmentation and proper message targeting [24]. Both factors might account for why we did not find a clear, persuasive advantage of our targeted messages. Sufficient homogeneity among members of a given subgroup is the premise of successful targeted communication [61]. The behavior domain of tobacco use, however, is usually identified as being associated with a large variability of behavioral determinants [62]. It may be possible that heterogeneity existed in our three smoker groups. In addition, although the current study considered several psychological and behavioral attributes when creating segments, it is possible that some key elements that were most crucial to produce distinctive changes were not included. For instance, previous studies have suggested that social support is an effective construct to segment audiences [63]. However, we did not incorporate that into our segmentation strategy. In addition, our targeted messages might not relate strongly to each group. Based on previous literature [63], adding more visuals and more customized elements specific to each segment might help increase the effectiveness of the targeted messages. Given that targeted communication has shown its potential to enhance health communication effectiveness in many domains and that smokers vary dramatically in their ENDS use patterns and risk perceptions, we urge more studies to continue exploring messages that target communication about ENDS with more convincing messages and more refined audience segmentation.

Findings of our study should be interpreted in light of several limitations. First, our study did not include a control group. Hence, we cannot make definitive claims about the actual effectiveness of targeted and nontargeted messages in changing people’s risk perceptions or intentions. Second, our study is limited by a single forced message exposure to the targeted message for each group, which limits the external validity of this study. In addition, we used self-reported behavioral intentions instead of people’s actual behaviors as the outcome of message exposure. Although intention is conceptualized as a proximal predictor of a person’s actual behavior, to better understand the impact of targeted vs. nontargeted messages on behavior, actual behavior should be measured in future studies. Finally, many outcome measures were assessed by a single question. Multi-item measures might better represent each concept and increase stability of the results.

## 5. Conclusions

Despite multiple limitations, our study is novel in examining audience segmentation and message targeting in the context of communication about the risk of ENDS, which extends the scholarship on strategic message customization. In addition, although our targeted messages did not show a persuasive advantage to communicate comparative ENDS risks, our study adds to the limited literature on the best way to communicate about ENDS. As there is an increasing demand for accurately informing the public of the risk differences associated with different tobacco products, our findings provide useful insights into this topic.

## Figures and Tables

**Table 1:** Characteristics of three different smoker groups identified in this project.

		Older Freedom Smokers	Reluctant Smokers	Young Enthusiasts
Inclusion Criteria	Age	45–64	30–55	18–30
	Education	N/A	N/A	At least some college education
	Income	<$45 k	<$55 k	>$30 k
	Tobacco Use	Have used ENDS and are currently using them once a month or less frequently	Want to quit smoking at least “a little” and have never used ENDS	Currently using ENDS at least three times per week or more
	Worldviews	Individualistic worldviews ^1^	N/A	N/A
Focus Groups Findings	ENDS Attitudes and Risk Perceptions	More likely than not to think of ENDS as equally or more dangerous than combusted cigarettes Do not consider ENDS as a way to quit cigarettes/concerned about using ENDS for quitting because it could lead to dependency on another product	Thought ENDS were equally or more dangerous than combusted cigarettes Do not consider ENDS as a way to quit cigarettes: unsafe, not natural, and contain dangerous chemicals	More likely than other groups to believe ENDS are less harmful than combusted cigarettes Most likely than other groups to see ENDS as a way to reduce combusted cigarette use Less likely than other groups to state that they wanted to stop their use of ENDS

^1^ Individualistic worldviews were defined as answering “yes” to both of these statements: “The government interferes far too much in our everyday lives” and “It’s not the government’s business to try to protect people from themselves”.

**Table 2:** Messages targeting three different smoker groups identified in this project.

**Message Targeting Older Freedom Smokers** You are in charge and you call the shots about how you want to live your life. One choice you’ve made is to smoke cigarettes and you would never want anyone to take this away from you. But perhaps the time has come for you to take it away from you. As the years go by, it gets harder to ignore the reality of what smoking is doing to your health. Shortness of breath is just the beginning. You know it will only get more serious. So before it gets any worse, now’s the time to quit and get your freedom back. Some smokers say using ENDS helped them quit combusted ones. There is research that says ENDS may be as effective as the nicotine patch for helping people quit smoking. If you’re thinking about quitting, perhaps with the help of ENDS, you don’t have to do it alone. Get support to quit smoking from your doctor, support group, family member, or friend.
**Message Targeting Reluctant Smokers** Heart disease, lung cancer, emphysema-smoking is incredibly harmful to your health. But for many of us smokers, just being aware of the dangers isn’t enough to help us stop. If you’re still searching for something that works for you, ENDS could be a renewed hope to help you quit. They are less harmful than traditional ones, and research has shown that they may be as effective as other nicotine replacement therapies, like the patch. ENDS still have other chemicals, so think of them as a short-term quit method—a temporary replacement for traditional cigarettes on your path to becoming completely tobacco-free. The best thing you can do is to have a plan to quit all nicotine products for good—one that includes support from a doctor, family member, or friend.
**Message Targeting Young Enthusiasts** You’re young, smart—a real original. You have so many cool experiences to look forward to in your life. Starting a family, following your muse, traveling the world. Are you ready to risk all of that for Unicorn Blood and Mother’s Milk? No matter the name or flavor, all vaping products contain chemicals, some of which may be harmful to you. They’re not as dangerous as combusted cigarettes. But what happens when you can’t quit them or even worse, when you use them and regular cigarettes—and can’t quit either? If you are only vaping to help you quit regular cigarettes—okay, just be sure you have a plan in place that includes support from a doctor, family member, or friend so you can ditch all vaping and smoked tobacco products and spend your life nicotine free and healthy.

**Table 3:** Effects of targeted and nontargeted messages on perceived comparative risk of ENDS.

Predictors	Less Harmful (vs. More or Equally Harmful or I Don’t Know) OR (95% CI)
Old Freedom Smokers (OFS) (Nagelkerke *R*^2^ = 0.57)	
OFS vs. RS message	1.38 (0.46, 4.12)
OFS vs. YE message	1.87 (0.65, 5.37)
Reluctant Smokers (RS) (Nagelkerke *R*^2^ = 0.47)	
RS vs. OFS message	2.44 (1.01, 5.89) *
RS vs. YE message	2.74 (1.08, 6.94) *
Young Enthusiasts (YE) (Nagelkerke *R*^2^ = 0.60)	
YE vs. OFS message	0.80 (0.26, 2.43)
YE vs. RS message	0.37 (0.11, 1.23)

*Notes.* Results of binominal logistic regression controlling for perceived absolute ENDS risk at pretest, perceived absolute cigarette risk at pretest, perceived comparative risk of ENDS to cigarettes at pretest, response efficacy of ENDS to help quit smoking at pretest, and response efficacy of ENDS to reduce smoking at pretest. * *p* < 0.05.

**Table 4:** Means and standard errors of targeted and nontargeted messages on ENDS- and cigarette-related beliefs and behavioral intentions.

	Old Freedom Smokers (OFS)			Reluctant Smokers (RS)			Young Enthusiasts (YE)		
	Targeted Message	Nontargeted Messages		Targeted Message	Nontargeted Messages		Targeted Message	Nontargeted Messages	
Dependent Variables	OFS Message	RS Message	YE Message	RS Message	OFS Message	YE Message	YE Message	OFS Message	RS Message
Perceived Absolute ENDS Risk	3.24 ^a^ (0.12)	3.17 ^a^ (0.11)	3.62 ^b^ (0.10)	3.26 ^a,b^ (0.13)	3.12 ^a^ (0.12)	3.55 ^b^ (0.13)	3.40 ^a^ (0.11)	2.96 ^b^ (0.12)	2.93 ^b^ (0.11)
Perceived Absolute Cigarette Risk	4.51 (0.11)	4.50 (0.10)	4.39 (0.09)	4.53 (0.11)	4.56 (0.10)	4.48 (0.10)	4.50 (0.08)	4.56 (0.08)	4.63 (0.08)
Response Efficacy of ENDS to Help Quit Smoking	3.49 ^a^ (0.11)	3.68 ^a^ (0.10)	3.11 ^b^ (0.09)	3.18 (0.12)	3.16 (0.11)	3.00 (0.12)	3.70 ^a^ (0.10)	4.23 ^b^ (0.10)	4.15 ^b^ (0.10)
Response Efficacy of ENDS to Reduce Smoking	3.68 ^a^ (0.11)	3.84 ^a^ (0.10)	3.28 ^b^ (0.09)	3.39 (0.14)	3.24 (0.12)	3.06 (0.13)	3.94 ^a^ (0.09)	4.30 ^b^ (0.09)	4.30 ^b^ (0.09)
Self-Efficacy	2.61 (0.11)	2.49 (0.10)	2.42 (0.09)	2.71 ^a^ (0.11)	2.76 ^a^ (0.10)	2.32 ^b^ (0.11)	3.11 (0.09)	3.37 (0.10)	3.31 (0.09)
Quit Intentions	3.89 (0.14)	3.90 (0.13)	3.91 (0.12)	4.37 (0.11)	4.23 (0.10)	4.22 (0.11)	3.80 ^a^ (0.11)	3.92 ^a,b^ (0.12)	4.23 ^b^ (0.11)

*Notes.* Results of analyses of covariance (ANCOVAs) controlling for perceived absolute ENDS risk at pretest, perceived absolute cigarette risk at pretest, perceived comparative risk of ENDS to cigarettes at pretest, response efficacy of ENDS to help quit smoking at pretest, and response efficacy of ENDS to reduce smoking at pretest. Different letters indicate significant differences at *p* < 0.017 (Bonferroni corrected error) based on pairwise comparisons following ANCOVAs.

**Table 5:** Means and standard errors of targeted and nontargeted messages on perceived message effectiveness and message reactions.

	Old Freedom Smokers (OFS)			Reluctant Smokers (RS)			Young Enthusiasts (YE)		
	Targeted Message	Nontargeted Messages		Targeted Message	Nontargeted Messages		Targeted Message	Nontargeted Messages	
Dependent Variables	OFS Message	RS Message	YE Message	RS Message	OFS Message	YE Message	YE Message	OFS Message	RE Message
Perceived Message Effectiveness ^i^	3.60 ^a,b^ (0.07)	3.70 ^a^ (0.06)	3.47 ^b^ (0.08)	3.82 ^a^ (0.06)	3.80 ^a^ (0.06)	3.59 ^b^ (0.07)	3.43 ^a^ (0.08)	3.80 ^b^ (0.06)	3.83 ^b^ (0.06)
Negative Emotions ^i^	1.65 (0.06)	1.74 (0.07)	1.62 (0.06)	2.05^a^ (0.08)	1.93 ^a,b^ (0.07)	1.83 ^b^ (0.07)	1.95 (0.07)	1.93 (0.07)	1.92 (0.08)
Positive Emotions ^i^	2.63 ^a^ (0.09)	2.63 ^a^ (0.09)	2.23 ^b^ (0.09)	3.12 ^a^ (0.09)	3.10 ^a^ (0.09)	2.59 ^b^ (0.09)	2.61 ^a^ (0.09)	3.23 ^b^ (0.09)	3.15 ^b^ (0.09)
Smoking Cravings ^ii^	3.46 (0.19)	3.26 (0.18)	2.92 (0.16)	3.00 (0.17)	2.94 (0.15)	2.74 (0.16)	2.56 (0.14)	2.72 (0.15)	2.89 (0.14)
ENDS Use Cravings ^ii^	2.67 ^a^ (0.14)	2.50 ^a^ (0.13)	2.03 ^b^ (0.12)	2.45 (0.14)	2.51 (0.13)	2.06 (0.14)	3.35 ^a^ (0.14)	3.83 ^b^ (0.15)	3.62 ^a,b^ (0.14)

Notes. ^i^ Results of repeated-measures analyses of variance (ANOVAs). Different letters indicate significant differences at *p* < 0.017 (Bonferroni corrected error) based on pairwise comparisons following repeated-measure ANOVAs. ^ii^ Results of analyses of covariance (ANCOVAs) controlling for perceived absolute ENDS risk at pretest, perceived absolute cigarette risk at pretest, perceived comparative risk of ENDS to cigarettes at pretest, response efficacy of ENDS to help quit smoking at pretest, and response efficacy of ENDS to reduce smoking at pretest. Different letters indicate significant differences at *p* < 0.017 (Bonferroni corrected error) based on pairwise comparisons following ANCOVAs.

## Supplementary Materials

The questions are available online at http://www.mdpi.com/1660-4601/15/10/2071/s1.
