# Supplementary material for: Targeted Versus Nontargeted Communication About Electronic Nicotine Delivery Systems in Three Smoker Groups
Source: Int J Environ Res Public Health. 2018 Sep 21;15(10):2071. doi: 10.3390/ijerph15102071 (PMC6210660; doi:10.3390/ijerph15102071)
Supplement: Supplementary file 1 [file ijerph-15-02071-s001.pdf]

QUESTIONS USED TO IDENTIFY THE THREE SMOKER GROUPS:

1. What is your current age? __________ years old

2. What is the highest level of education you have completed?

Some high school	[ ]
High school graduate or GED	[ ]
Some college, vocational or technical school	[ ]
College graduate or more	[ ]

3. What is your total annual household income?

Less than $25,000	[ ]
$25,000 to $44,999	[ ]
$50,000 to $74,999	[ ]
$75,000 or more	[ ]

4. Please indicate which statements you agree with.

The government interferes far too much in our everyday lives.	[ ] Yes<br>[ ] No
It's not the government's business to try to protect people from themselves.	[ ] Yes<br>[ ] No

5. Do you currently smoke traditional cigarettes every day, somedays, or not at all?

Everyday	[ ]
Somedays	[ ]
Not at all	[ ]

6. Have you ever used electronic vapor products, even one or two times?

No…………………..	[ ]
Yes ………………..	[ ]

7. How often do you currently use electronic vapor products?

A few times per year	[ ]
Once a month	[ ]
Once a week	[ ]
At least three times per week or more	[ ]

8. How much do you want to quit smoking? Would you say you want to quit…

Not at all	[ ]
A little	[ ]
Somewhat	[ ]
A lot	[ ]
